# PEP Does Not Dispense with but Implements Task-Set Reconfiguration. Can It Handle Phenomena More Diagnostic of Endogenous Control?

**DOI:** 10.5334/joc.109

**Published:** 2020-09-10

**Authors:** Stephen Monsell, Ian McLaren

**Affiliations:** 1 University of Exeter, GB

**Keywords:** Cognitive Control, Executive functions, Task-switching

## Abstract

Invited commentary on Schmidt, Liefooghe, and De Houwer (2020) An episodic model of task switching effects: erasing the homunculus from memory. *Journal of Cognition*.

In their article, Schmidt, Liefooghe, and De Houwer (2020) — henceforward “SL&DH”:

1. focus on how immediate repetition of cues, stimuli and responses in task-cuing experiments modulate RT and error rate, inflating (they say) the “true” cost of a task switch;
2. propose a computational account — PEP — in terms of retrieval of episodic traces in which stimulus, cue and response representations have been bound to tasks initially by instruction, then by recent trials;
3. argue that the PEP model is sufficient to explain the phenomena observed in task- switching experiments phenomena without appeal to control via a task-set reconfiguration (TSR) control process.

Deconstruction of quasi-homuncular theoretical constructs of executive control in terms of an “army of idiots” — simple well-specified mechanisms, such as memory retrieval, that we think we understand — is a goal we share with SL&DH (Monsell & Driver, 2000; Verbruggen, McLaren & Chambers, 2014). We commend their explicit modelling of the influence of immediate repetitions on the switch cost, but comment here, in turn, on these three components of their argument, of which the third is the most contentious.

## Effect of immediate repetitions and the switch cost

It has been known for a long time that task-switch costs are modulated by immediate repetition of cues (Logan & Bundesen, 2003), and responses (Rogers & Monsell, 1995) and that immediate repetition of a stimulus enables abbreviated processing on task-repeat trials (Bertelson, 1961, Kornblum, 1973), while inducing interference when the task changes. SL&DH model data from a study by Schmidt and Liefooghe (2016), which carefully assessed how all three kinds of repetition modulated the task-switch cost, finding the effects to be substantial. However, the assertion on the basis of just one study that, in the absence of all three kinds of immediate repetition, “true” switch costs are relatively trivial is surely premature.^1^ As SL&DH acknowledge, substantial switch costs have been observed in numerous experiments where cue repetitions have been prevented, and/or large stimulus or response sets have been used so that repetitions occured, if at all, at long lags.

## Explanation of immediate repetition effects

Associative “binding” or “integration” in episodic traces is certainly a candidate mechanism to explain effects of repetition. The model fits render it plausible. But there are other accounts of immediate repetition effects, notably priming, as in the Schneider and Logan (2005) explanation of cue repetition effects as priming of cue encoding due to residual activation. We add the terminological note that even if one hypothesizes that an effect results from binding or feature integration biases, one should not slip into calling these effects “binding” or “integration” effects: this muddles description and interpretation.^2^

Perhaps because their focus is on effects of immediate repetition, SL&DH seem to make no distinction between transient binding in event files in working memory (e.g. Hommel, 1998) and long term learning of associations, as in the association of task-sets to stimuli proposed by Waszak, Hommel and Allport (2003). And the attribution of associative effects to episodic instance representations rather than more abstractive associative learning seems — as in other domains of cognition — an ideological rather than an evidence-determined choice.

## Does the PEP model dispense with TSR?

SL&DH’s radical claim is that the PEP model provides a simpler, mechanistic, alternative to a TSR account of task-cuing and -switching. In thus reaching for Occam’s razor, we think SL&DH over-reach. First, the focus of their modelling is on modulations of the switch cost by repetition effects and the effect of response congruence. The empirical phenomena that primarily motivate a TSR account are mentioned only as an afterthought, with the hope that the PEP model may be able to handle them. Second, the TSR account which SL&DH reject (“it seems clear that the PEP model is not consistent with the task-set reconfiguration account”) is a straw man. Indeed, as far as we can see, their PEP model *implements* an appropriately formulated TSR account.

The observation most suggestive of an active TSR process is that motivated participants can achieve a substantial reduction of switch cost (“RISC”) if given foreknowledge (e.g. by a task cue) of the task to be required when the stimulus appears, and time to prepare. No less important is the asymptotic “residual cost” seen after a long preparation interval: this implies limits to what can be achieved by anticipatory TSR.

In the PEP model, goal nodes, their connections, and episodic traces created initially by instruction, represent the task-sets in play; task-sets are selected by activation of the goal nodes. SL&DH think this “overstates [the] function” of the goal nodes, because “all these nodes do is retrieve the viable decision options it is considering”. But, once the network is set up and instructed by seeding episodic traces, that is all the task-set control needed for their paradigm. Cues activate the task nodes, which then modulate the response activation produced by the stimulus (to a degree biased by recent episodic bindings) in an explicitly two-step process: task-set selection, then response selection. The first step *is* the reconfiguration process. This may be contrasted with Schneider and Logan’s (2005) one-step compound stimulus model, which really does dispense with control of task set (albeit, we think, inappropriately; see Forrest, Monsell & McLaren, 2014).

SL&DH think their PEP model is inconsistent with a TSR account because they assume that TSR must be a discrete stage rather than a dynamic process, and requires a “switch detector” to initiate task preparation on switch trials, with no preparation on repeat trials. Neither is a necessary property of the broad class of TSR accounts. While some early variants did construe TSR as a discrete stage (or two stages, e.g. Rogers & Monsell, 1995), or an all-or-none accomplishment (De Jong, 2000), in more recent discussions we (e.g. Monsell, 2015, 2017; Monsell & Mizon, 2006) and others acknowledge the possibility of TSR being an incremental dynamic process (as in connectionist characterisations of control of task-set by context, e.g. Miller and Cohen, 2001; Gilbert and Shallice, 2002) admitting of degrees of accomplishment. Research on the “posterior positivity” believed to be an ERP correlate of anticipatory TSR has highlighted trial to trial variability in the degree of preparation achieved (Lavric, Mizon & Monsell, 2008; Karayanidis et at, 2011). Nor is a “switch detector” essential to the TSR account (though some authors have assumed or implied one.) Strictly speaking, in TSR accounts “reconfiguration” is not the control *process* but its *result* (on switch trials). The “act of control” — activation of the task-set triggered by the cue (or other foreknowledge) — may well happen on all trials, as in the PEP account. *Re*configuring of task-set links and parameters is the result achieved (or at least attempted) by activating the cued task-set on switch trials; preparatory (re)activation of the current task-set may indeed occur on repeat trials as well, reinforcing the existing configuration (see Steinhauser, Maier and Ernst, 2017, for ERP evidence).

As far as we are aware, no recent theorist attributes switch costs solely to TSR; most accounts are “hybrid” (Monsell, 2003) in the sense that the switch cost, the RISC function, and response congruence effects, are the outcomes of, essentially, a competition between the control process and what it has to overcome, namely carry over of the previous tasks’ parameters (orientation of attention, S-R rules, etc) or inhibition thereof (Allport, Styles & Hsieh, 1994), and/or associative reactivation by the stimulus of task-set parameters (Waszak, Hommel & Allport, 2013). (For review see Kiesel et al., 2010; Monsell, 2015; Vandierendonck, Liefooghe, & Verbruggen, 2010). The switch cost indexes not the duration of TSR but its efficacy. The rate at which the switch cost reduces as the preparation interval increases (the slope of the RISC function) is as close as we can get to timing a TSR process using RT. In their Discussion, SL&DH speculate that the PEP model may explain the effect of preparation interval by assuming early activation of the goal nodes. This is anticipatory TSR! It remains to be seen whether a parameterisation of the PEP model which can simulate the RISC effect will also simulate the residual cost.

An apparent virtue of the PEP account is that, once the network is set up and episodic traces formed, performance is driven automatically by the cue and stimulus on each trial, biased by the recent past, without endogenous control exterior to the network. Indeed, there is no distinction between endogenous and exogenous control; all the task-set control needed is exercised via activation of goal nodes by cues. As a consequence, the PEP model seems ill-equipped to deal with the variable and volitional quality of task-set control. Participants do not really *have* to proactively prepare in most experiments with a long preparation interval, and a RISC effect is sometimes not seen under conditions that we may suspect demotivate advance preparation (e.g. Altmann, 2004; Roger & Monsell, 1995). Switch costs exhibit substantial variability both over trials and over participants in a way most easily understood as variability in TSR effort, not just noise. The timing of the ERP posterior positivity is sensitive to when the stimulus is expected, not simply time-locked to the cue or previous response (Karayanidis et al., 2003, 2014). Participants appear to adjust how fully or often they prepare for a change of task as a function of the probability (Monsell & Mizon, 2006; Mayr, Kuhns & Rieter, 2013) or predictability (Monsell, Sumner & Waters, 2003) of a task-switch in the immediate future. In short, on top of the control already embedded in the PEP model, it would appear to require a superordinate level of monitoring and adjustment of the exercise of that control (as in the Brown, Reynolds & Braver, 2007, network).

Moreover, in experiments very like Schmidt and Liefooghe (2006), but with only 4 stimuli, Forrest et al. (2014) found that participants could be induced to adopt, and sometimes discovered without explicit instruction, Schneider and Logan’s (2005) one-step strategy of retrieving responses to cue-stimulus compounds. This manifested in a pattern of performance (minimal switch costs, no RISC effect, and large congruence effects modulated by practice) very different from the standard task-switching pattern obtained in participants given standard task-cuing instructions and identical cue-stimulus trial sequences. A PEP network might well be able to simulate this alternative pattern, but would still need an account of how two such different patterns of performance could emerge with identical sequences of cues and stimuli.

Hence, far from “erasing” task-set reconfiguration, SL&DH’s PEP model implements a minimal version of it, but needs to be tested for its ability to handle the RISC function, including the residual cost, and will need additional mechanisms to capture phenomena suggestive of strategic modulation of control and process architecture.

## Footnotes

Cf. Logan and Bundesen’s (2003) initial claim that most of the task-switch cost in cueing experiments could be accounted for by a confound of task- and cue-repetition; although cue repetition is a significant factor, robust switch costs have generally been found when cue repetition is controlled or prevented (e.g. Monsell & Mizon, 2006).

Cf. the labelling of instances of *interference* (with performance) as cases of *inhibition* when the latter is only one possible mechanism for the former (MacLeod et al., 2003). For the same reason, in task-switching we should not call n-2 repetition benefits “backward inhibition”.
